# Tula hantavirus isolate with the full-length ORF for nonstructural protein NSs survives for more consequent passages in interferon-competent cells than the isolate having truncated NSs ORF

**DOI:** 10.1186/1743-422X-5-3

**Published:** 2008-01-11

**Authors:** Kirsi M Jääskeläinen, Angelina Plyusnina, Åke Lundkvist, Antti Vaheri, Alexander Plyusnin

**Affiliations:** 1 Department of Virology, Haartman Institute, PO Box 21, FIN-00014 University of Helsinki, Helsinki, Finland; 2 Swedish Institute for Infectious Disease Control, S-171 82 Stockholm, Sweden; 3 Department of Microbiology, Tumor and Cell Biology, Karolinska Institutet, S-171 77 Stockholm, Sweden

## Abstract

**Background:**

The competitiveness of two Tula hantavirus (TULV) isolates, TULV/Lodz and TULV/Moravia, was evaluated in interferon (IFN) -competent and IFN-deficient cells. The two isolates differ in the length of the open reading frame (ORF) encoding the nonstructural protein NSs, which has previously been shown to inhibit IFN response in infected cells.

**Results:**

In IFN-deficient Vero E6 cells both TULV isolates survived equally well. In contrast, in IFN-competent MRC5 cells TULV/Lodz isolate, that possesses the NSs ORF for the full-length protein of 90 aa, survived for more consequent passages than TULV/Moravia isolate, which contains the ORF for truncated NSs protein (66–67 aa).

**Conclusion:**

Our data show that expression of a full-length NSs protein is beneficial for the virus survival and competitiveness in IFN-competent cells and not essential in IFN-deficient cells. These results suggest that the N-terminal aa residues are important for the full activity of the NSs protein.

## Background

Hantaviruses (genus *Hantavirus*, family *Bunyaviridae*) are carried by rodents and insectivores and present all over the world [1]. Some hantaviruses are nonpathogenic, and others are human pathogens. Pathogenic hantaviruses from Asia and Europe cause hemorrhagic fever with renal syndrome (HFRS) while hantaviruses in the Americas cause hantavirus pulmonary syndrome (HPS). The genome of hantaviruses consists of three segments of a negative-sense single-stranded RNA. The large (L) segment codes for RNA polymerase (L protein), the medium (M) segment for two glycoproteins Gn and Gc, and the small (S) segment for the nucleocapsid (N) protein [1]. Hantaviruses carried by Cricetidae rodents (subfamilies Arvicolinae, Neotominae, and Sigmodontinae) have in their S segment an additional +1 open reading frame (ORF) for the nonstructural protein NSs [2]. Hantaviruses carried by Muridae rodents (subfamily Murinae) do not possess the NSs ORF [2]. Most recently, we have shown that the hantaviral NSs protein is an inhibitor (albeit not a strong one) of the interferon (IFN) response [3].

The IFN response is one of the main host defence mechanisms against viruses. Virus infection induces expression of several IFN genes, in most cell types first the genes encoding IFN-β and IFN-α4 [4]. These IFN proteins are then secreted from an infected cell and they bind to corresponding receptors on the same or neighbouring cells starting a signaling cascade that leads to expression of hundreds of IFN-stimulated genes producing powerful antiviral proteins such as myxovirus resistance gene (Mx), 2'–5' oligoadenylate synthetases (OAS) and protein kinase stimulated by dsRNA (PKR) (reviewed in [5]). Many viruses have developed special mechanisms to evade the host immune response (for a review, see [6,7]). For example, orthobunyaviruses and phleboviruses from the *Bunyaviridae *family encode NSs proteins that inhibit the host cell immunity by suppressing host transcription [8-11]. Our previous data show that the NSs ORF in *Tula *(TULV) and *Puumala *(PUUV) hantaviruses is functional [3]. TULV NSs protein was seen with coupled *in vitro *transcription and translation from S segment cDNA. PUUV NSs protein was seen with Western blot in infected Vero E6 cells. Transiently expressed NSs proteins of both TULV and PUUV inhibited the activities of IFN-β promoter, and nuclear factor kappa B (NF-κB)- and interferon regulatory factor-3 (IRF-3) responsive promoters in COS-7 cells. The decline in the expression of IFN-β mRNA was evident in TULV- infected or TULV- NSs expressing IFN-competent MRC5 cells. These data strongly suggested that the hantaviral NSs protein is an IFN antagonist.

**Figure 1:**
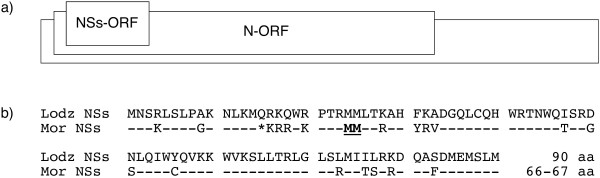
**Hantavirus NSs ORF**. a) Schematic presentation of hantavirus S segment. TULV NSs protein is 90 aa and N protein 429 aa in length. b) NSs ORF sequences of TULV/Lodz and TULV/Moravia. TULV/Lodz codes for the full-length NSs protein of 90 aa. TULV/Moravia NSs ORF contains a stop codon at the place of Glu-15 and the production of truncated protein presumably begins from Met-24 or Met-25 (bold and underlined) and thus yields a protein of 66–67 aa in length. *, stop codon.

In this study we aimed to find whether the length of the NSs ORF can affect the hantavirus capacity to withstand the host IFN response. We took advantage of the availability of two TULV isolates, TULV/Lodz [12] and TULV/Moravia [13]. These two TULV isolates differ in the length of the NSs ORF. In TULV/Lodz the NSs ORF is 90 aa long while in TULV/Moravia a single mutation generated during adaptation to Vero E6 cell culture converted the 15th triplet into a stop codon (Fig. 1). Consequently, this isolate produces a slightly shorter NSs protein of 67–68 aa residues, which most probably starts from Met24 or Met25 [3] (Fig. 1). IFN-competent MRC5 cells [14] and, as control, IFN-deficient Vero E6 cells [15] were infected with a mixture of the viruses and isolate-specific RT-PCR assays were utilized to find out, which of the two isolates resists the IFN response better.

## Results

### Selection of primers for isolate-specific amplification of the S and M segment sequences of two TULV isolates

**Figure 2:**
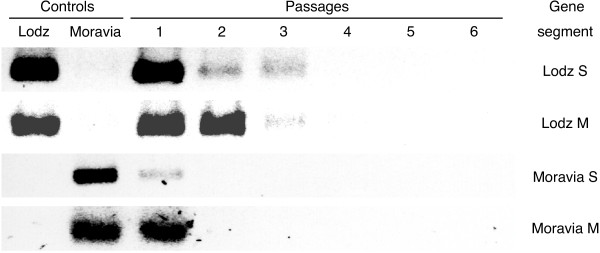
**Detection of TULV/Lodz and TULV/Moravia S and M segments in double-infected MRC5 cells**. Cells were infected with the mixture of the TULV strains; fresh cells were infected with supernatant, and the cells were used for RNA isolation. RT-PCR was performed with isolate- and gene-specific primers. From up: results of RT-PCR assays with the primers specific for: TULV/Lodz S segment, TULV/Lodz M segment, TULV/Moravia S segment, and TULV/Moravia M segment.

**Figure 3:**
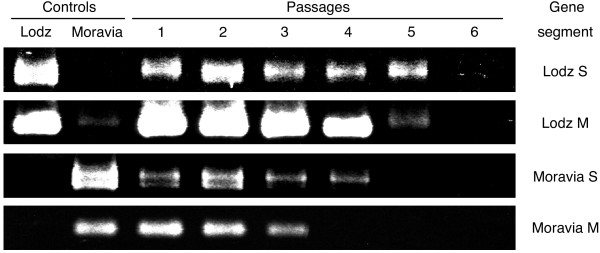
**Detection of TULV in MRC5 cells infected with the supernatant from double-infected Vero E6 cells**. MRC5 cells were infected with the passage 1 supernatant from Vero E6 cells infected with the mixture of TULV/Lodz and TULV/Moravia. Supernatant was used to infect fresh cells, and from them RNA was isolated. RT-PCR was done with the isolate-specific S- and M-primers. From top: results of RT-PCR assays with the primers specific for: TULV/Lodz S segment, TULV/Lodz M segment, TULV/Moravia S segment, and TULV/Moravia M segment.

First, we designed isolate-specific primers for detection either of two TULV isolate during double infection. As the isolates are genetically closely related only a few potential regions for the annealing of isolate-specific primers could be found in their genomes. Our S-primers appeared isolate-specific indeed (Figures 2 and 3) and allowed to amplify 266 bp and 255 bp products from TULV/Lodz and TULV/Moravia isolates, respectively. RT-PCR assays with these S-primers appeared also quite sensitive: the PCR-products were seen after 30 rounds of amplification. The selected M-primers showed the high specificity as well but, to generate sufficient amount of amplicons, nested PCR was needed (Table 1).

**Table 1:** Primers used in TULV isolate-specific RT-PCR assays.

Primer name (isolate, segment, forw/rev)	Sequence 5'-3'	Position (nt)	Amplicon size (bp)
LVSF783 (Lodz, S, forw)	GAAAAAGCAAGGTGGTCCCAAC	783–804	266
LVSR1026 (Lodz, S, rev)	GGATTGAGAAGAAGGCTCCTAAT	1026–1048	
LodzG2F426 (Lodz, M, for)	CAAATTGAGGTCAGTCGGG	426–444	528
LodzG2R953 (Lodz, M, rev)	AATGATAAATCCCTATTGACG	933–953	
LodzG2F554 (Lodz, M, nested PCR, forw)	CCGTTAAAGTTTGCATGATAGGG	554–576	261
LodzG2R814 (Lodz, M, nested PCR, rev)	GTTGATAGCCAGAAACTGTATTG	792–814	
TulSF895 (Moravia, S, forw)	GATTGATGACTTGATTGATCTTGC	895–918	255
MVSR1149 (Moravia, S, rev)	GCGTCTCAGATATGACTGATAG	1128–1149	
MorG2F83 (Moravia, M, forw)	CTGATTTAGAATTGGATTTTTCCC	83–106	735
MorG2R817 (Moravia, M, rev)	TTCTCTGATATCCAGATACAGTG	795–817	
MorG2F444 (Moravia, M, nested PCR, forw)	CAAAGTTTATAAAATCCTGTCCC	444–466	136
MorG2R579 (Moravia, M, nested PCR, rev)	TGTTCCAATCATACAGACCTTC	558–579	

### Survival and competitiveness of TULV isolates in IFN-deficient cells

Vero E6 cells were infected with a mixture of TULV/Lodz and TULV/Moravia isolates. After 14 days fresh Vero E6 cells were infected with a part of the supernatant and again new supernatant was collected and used in infection (for details, see Methods). Altogether 10 passages were performed. Total RNA was isolated from infected cells and the isolate-specific RT-PCR assays were used to monitor the presence of viral S and M segments. The S- and M- amplicons of both isolates were seen during all passages (Table 2), i.e. none of the viruses outcompeted another. These results suggested that, at least under these experimental conditions, the length of the NSs protein did not affect the competitiveness of the virus in IFN-deficient cells. When the mixed infection was repeated in IFN-competent cells, the situation changed.

**Table 2:** Summary of RT-PCR detection of TULV S and M segment RNA.

			Passages									
			
Cells	Infection with TULV isolates	TULV isolate	1	2	3	4	5	6	7	8	9	10
Vero E6	Lodz & Moravia	Lodz	+	+	X^a^	+	+	+	+	+	+	+
		Moravia	+	+	X	+	+	+	+	+	+	+
MRC5	Lodz & Moravia	Lodz	+	+	+	-	-	-	ND^b^	ND	ND	ND
		Moravia	+	-	-	-	-	-	ND	ND	ND	ND
MRC5	1st passage from VeroE6	Lodz	+	+	+	+	+	-	ND	ND	ND	ND
		Moravia	+	+	+	+/- ^c^	-	-	ND	ND	ND	ND

^a^RNA pellet from passage 3 was lost and therefore we were unable to detect viruses in this passage;

^b^ND = not done;

^c^The S-specific RT-PCR was positive up to passage 4; the M-specific RT-PCR was positive up to passage 3.

### Survival and competitiveness of TULV isolates in IFN-competent cells

MRC5 cells were infected with the mixture of TULV/Lodz and TULV/Moravia isolates. The supernatant was collected and used to infect fresh cells. Altogether 6 passages were performed and the RNA was analyzed by RT-PCR assays. While both S and M segments of TULV/Lodz were detected during three passages, the corresponding segments of TULV/Moravia were detected only at passage 1 (Fig. 2). When MRC5 cells were infected with the first passage supernatant from Vero E6 cells infected with the mixture of two viruses, the outcome was essentially the same (Fig. 3). Neither of the isolates survived all six passages, and the TULV/Lodz isolate probably producing 90 aa-long NSs protein survived better than TULV/Moravia isolate capable of producing a shorter version of the NSs protein. Interestingly, under these experimental settings both TULV isolates survived better.

## Discussion

IFN response plays an important role during hantavirus infection [16-21] and, not surprisingly, hantaviruses replicate better in IFN-deficient than in IFN-competent cells [19,22,23].

NSs ORF is found in many but not in all hantaviruses [2]. Both nonpathogenic hantaviruses (e.g. TULV and *Prospect Hill virus*) and pathogenic ones (e.g. *Sin Nombre virus *(SNV) and *Andes virus*) have NSs ORF, and presumably produce the NSs protein. Thus this protein is probably not the sole determinant of hantavirus pathogenicity. An NSs ORF is present also in the S segments of bunyaviruses of the genera *Orthobunyavirus*, *Tospovirus*, and *Phlebovirus *[1]. The NSs proteins of orthobunya- and phleboviruses counteract the IFN response by inhibiting RNA polymerase II and hence downregulate the general transcription in infected cells [8-11]. By analogy one would assume a similar anti-IFN function for hantaviral NSs protein. According to our data, host protein synthesis is not severely affected by infection with TULV and PUUV. The NSs proteins of these viruses decrease the IFN response by inhibiting the activation of IFN-β promoter via NF-κB and IRF-3 pathways [3]. Thus the suppression of IFN-β induction by TULV, PUUV, and also Prospect Hill virus, *New York virus*, SNV, and Andes virus reported by several research groups [17-21] could be, at least in part, attributed to the inhibitory activity of the NSs protein. In hantaviruses lacking the NSs ORF, the IFN response could be antagonized by other means, e.g. by glycoproteins [21,23].

Here we have studied the competitiveness of two TULV isolates, TULV/Lodz and TULV/Moravia, after double infection in IFN-deficient and IFN-competent cells. These two TULV isolates differ in the length of their NSs ORF, which provided an opportunity to gain insights on function(s) of the NSs protein *in vivo*. TULV/Lodz isolate was expected to be more resistant to the IFN response than TULV/Moravia. This appeared to be the case indeed, supporting our earlier conclusion that the NSs protein is involved in the counteraction of IFN response, and suggesting that the N-terminal aa residues in the molecule are needed for the full activity of the NSs protein of TULV. It would be interesting to examine the anti-IFN activity of the NSs proteins of other hantaviruses, especially of SNV and SNV-like viruses that possess shorter NSs ORFs than PUUV and TULV [2].

Interestingly, even the more resistant of two TULV isolates, TULV/Lodz, failed to survive in MRC5 cells for more than five consequent passages. This temporary survival is in sharp contrast to the persistent, life-long infection, which TULV causes in its natural rodent host [24,25]. One possible explanation is that, in the course of natural infection, the virus infects only a few IFN-competent cells and thus can avoid an immediate clearance by the host innate immunity. In Vero E6 cells the full-length NSs protein of TULV/Lodz did not appear beneficial for the competitiveness of this isolate suggesting that the full-length NSs protein is not essential for the virus in IFN-deficient cells.

So far no hantavirus with the entire NSs ORF deleted has been found in nature or engineered using reverse genetics. However, an interesting clone of PUUV strain Sotkamo was recently obtained by focus purification technique from the original Vero E6 cell culture isolate [26]. This clone, Sotkamo-delNSs, carries a stop codon instead of Trp-21 codon in the NSs ORF, and thus could produce a truncated NSs protein (transcription presumably starts from Met-24), which is of the same size as in TULV/Moravia isolate. Most notably, Sotkamo-delNSs clone grows to substantially lower titers (about 10 times) than parental virus in IFN-competent A549 cells while in IFN-deficient Vero cells both viruses replicated with the same efficacy (Andreas Rang, personal communication). This is in agreement with our results on TULV and supports the idea that the production of the full-length NSs protein is beneficial for the viral growth in IFN-competent cells but not vital in IFN-deficient cells.

Reassortant variants could have been formed in the course of double infection with two TULV isolates. One could also assume that the reassortants possessing the S segment of TULV/Lodz isolate would have higher chances to survive in MRC5 cells (provided that the full-length NSs protein is a potent pro-survival factor). Unfortunately, our current isolate-specific RT-PCR assays are not quantitative and thus this hypothesis could not be properly evaluated. We are currently trying to develop real-time PCR assays to clarify this issue.

## Conclusion

The data presented here show that TULV/Lodz survives better in IFN-competent MRC5 cells than TULV/Moravia. This is probably due to the function of NSs protein, which in the former isolate is full-length while in the latter truncated and hence less active. The results are in agreement with our earlier findings on the anti-IFN function of TULV NSs protein [3]. The production of the full-length or truncated NSs protein appeared to have no effect on the competitiveness of TULV isolates in Vero E6 cells suggesting that in IFN-deficient cells the full-length NSs protein is not essential for virus growth.

## Methods

### Cells and viruses

Vero E6 cells were cultured in modified Eagle's medium (MEM) and MRC5 cells in Dulbecco's modified Eagle's medium (DMEM) with 10% fetal calf serum (FCS), 2 mM L-glutamine, penicillin and streptomycin in 5% CO_2 _at 37°C. TULV strain Lodz [12] and the cell culture-adapted isolate of TULV strain Moravia Tula/Moravia/Ma5302V/94 [13] were used.

### Titration of viruses

Confluent Vero E6 cells grown on 6-well plate wells were infected with several virus dilutions (0.5 ml) for 1 h. About 5 ml of 42°C 0.5% agarose, 8% FCS, 20 mM HEPES, 1 mM -glutamine, penicillin and streptomycin in MEM was added onto the cells. The plate was incubated for 10 min at room temperature (RT). After 11 days of incubation at 37°C the cells were fixed with 10% formaldehyde for 30 min at RT. Agarose was removed and cells were washed three times 5 min with 0.15% Tween-20 in PBS. The antibody reaction was done at RT for 1 h with 1% human anti-PUUV serum in 5% FCS, 0.15% Tween-20 in PBS. After washes, conjugate incubation was done at RT for 1 h with peroxidase-conjugated rabbit anti-human IgG diluted 1:150 in 0.15% Tween-20 in PBS. After washing, cells were stained with Liquid DAB+ Substrate Chromogen System (DakoCytomation, Glostrup, Denmark) according to the manufacturer's instructions. The titer was calculated by dividing the number of foci from a well having 2–5 foci, by the amount of virus put onto the cells.

### Double infections

About 80% confluent MRC5 cells grown on 25 cm^2 ^flasks were infected with TULV/Lodz and TULV/Moravia for 1 h (both MOI 0.2). The virus inoculum was then replaced with 10 ml DMEM. After 7 days of infection the supernatant (approximately 10 ml) was collected and the part of it (2 ml) was used to infect new cells. The remaining infected cells were used for RNA isolation. Consequently, the passage 2 supernatant was used to infect fresh cells 7 days post infection. Altogether 6 passages and samples for RNA isolation were collected. Confluent Vero E6 cells grown on 25 cm^2 ^flasks with medium containing 5% serum were infected with TULV/Lodz and TULV/Moravia (both 800 FFU). Lodz-Moravia passage 1 supernatant and samples for RNA isolation were collected 14 days post infection. New Vero E6 cells were infected with 1 ml of passage 1 supernatant with 9 ml medium containing 2% serum. After 14 days passage 2 samples were collected and fresh cells were infected with it. Totally 10 passages and samples for RNA isolation were assembled. The first passage of TULV/Lodz and TULV/Moravia mixed infection supernatant collected from Vero E6 cells was also used to infect MRC5 cells like above (MOI 0.04).

### RNA isolation

Cells from a 25-cm^2 ^flask were suspended to 3 ml of TriPure Isolation Reagent (Roche, Basel, Switzerland). RNA was isolated essentially according to the manufacturer's recommendation. Before use, the RNA was re-precipitated twice with ethanol and 3 M Na-acetate pH 5.3. RNA was dissolved in 25 μl H2O.

### RT-PCR

Reverse transcription was performed with 5 μl RNA and strain-specific primers using the SuperScript™ First-Strand Synthesis System for RT-PCR (Invitrogen, Carlsbad, CA) following the manufacturer's instructions. PCR was done with AmpliTaq^® ^DNA Polymerase (Applied Biosystems, Foster City, CA) with 5 μl cDNA, which was amplified with 250 μM dNTPs, 4 mM MgCl_2_, 1 μM of primers, and 0.03 U/μl polymerase. The isolate-specific primers are listed in Table 1. For Vero E6 samples Moravia S-segment PCR was done with the following primers [3]: forward MVSF780 5'-CCTGAAGAAAAGTGGTCCTAGT-3' and reverse MVSR1149 (Table 1). Later it was noticed that primer TulSF895 worked better together with MVSR1149 and this pair of primers was used in the amplification of MRC5-cell samples (Table 1). Due to the low sensitivity of the amplification of the M-segment sequences, the nested PCR was needed. PCR-amplicons were analyzed in 1.7% agarose gels.

## Competing interests

The author(s) declare that they have no competing interests.

## Authors' contributions

KMJ carried out most of the experiments and drafted the manuscript. AngP helped in RNA isolations and RT-PCR assays. ÅL and AV participated in drafting the manuscript. AP designed the study and participated in drafting the manuscript. All authors read and approved the final manuscript.
